# Influence of Bile Salts and Pancreatin on Dog Food during Static In Vitro Simulation to Mimic In Vivo Digestion

**DOI:** 10.3390/ani12202734

**Published:** 2022-10-11

**Authors:** Kangmin Seo, Hyun-Woo Cho, Jung-Hwan Jeon, Chan Ho Kim, Sejin Lim, Sohee Jeong, Kihyun Kim, Ju Lan Chun

**Affiliations:** Animal Welfare Research Team, National Institute of Animal Science, Rural Development Administration, Wanju-gun 55365, Korea

**Keywords:** beagle dog, static in vitro digestion model, bile salts, pancreatin, digestibility

## Abstract

**Simple Summary:**

Animal experiments are limited owing to concerns such as the ethics of animal use. To address the issue, an in vitro model for the stimulation of digestibility has been proposed. In the model, the physiological conditions of the oral cavity, stomach, small intestine, and ileum are selectively simulated, requiring careful consideration of the physiological parameters in each step. However, to date, the physiological roles and actions of digestive enzymes during the in vitro digestion of food ingredients remain unclear. In the present study, the effects of pancreatin and bile salts, which are the main digestive enzymes of the digestive tract, were evaluated. During in vitro digestion, the digestibility of crude protein, fat, and dry matter were influenced by the addition of various concentrations of pancreatin and bile salts. Therefore, the concentrations of pancreatin and bile salts should be carefully considered when applied in a static in vitro digestion model.

**Abstract:**

The addition of pancreatin and bile salts in different concentrations during in vitro digestion causes changes in the digestibility of crude protein (CP), fat, and dry matter (DM). The effects of bile salts and pancreatin on the digestibility of ether extract (EE), CP, and DM in developing a static in vitro digestion model for dogs were assessed using different concentrations of pancreatin (0, 1, 2.5, 5, and 10 g/L digestive solution) and bile salts (0, 2.5, 6.25, 12.5, and 25 g/L digestive solution). The data were analyzed using one-way analysis of variance. Digestibility of EE increased with the addition of bile salts (*p* < 0.05), whereas that of CP decreased with ≤0.25 g (1.0 g/L digestive solution) pancreatin. The digestibility of DM decreased significantly in all groups supplemented with ≥3.125 g (12.5 g/L digestive solution) bile salts and 0.25–2.5 g (1–10 g/L digestive solution) pancreatin and was the lowest with 6.25 g (25 g/L digestive solution) of bile salts (*p* < 0.05). These findings could facilitate the development of effective static in vitro digestion models for dogs.

## 1. Introduction

Active competition in the pet food market has led to the indiscriminate introduction of novel ingredients that have not been verified for safety or nutritional value in pets [1]. Although the ingredients are nutritionally exceptional and functional in humans, confirming their safety and functionality in pets is essential [2]. However, this requires significant economic investment and experimentation with animals, thereby hindering the development of feed/ingredients suitable for pets.

Animal research has been controversial owing to ethical concerns along with the reliability of animal experiments [3]. Reduction, refinement, and replacement (3R) of animal experiments have been the major strategy in research fields regarding animal ethics [4]. In an effort to support 3R, an alternative method has been encouraged [5]. As an alternative to in vivo studies, in vitro simulation of digestion has been used to analyze digestibility. An in vitro digestion model mimics the digestion process of an in vivo system and has advantages such as reproducibility and controlled selection of conditions. The final reactant can be easily collected and facilitates the assessment of bio-accessible fractions (absorbable nutrients) and predicts digestibility [6,7,8]. Therefore, it has become a popular alternative to ethically controversial animal testing in the field of food and nutrition. Static in vitro digestion models are commonly used, in which the ratio of food and enzyme is constant at each digestion step. Sophisticated computer-aided in vitro digestion models are capable of simulating physical or physiological aspects, such as the movement of digested food, real-time enzyme concentrations, and pH changes [9,10]. The in vitro simulation of digestion that mimics the physiology of the digestive system in humans is used to study the digestion and absorption of micronutrients with functional properties and major nutrients (e.g., proteins, carbohydrates, and fats) contained in various food types [11,12,13,14,15,16].

Commercial pet food contains over 10% crude fat [17], which is more than that in pig feed [18]. Previously, in animal research, pigs were the most used animals to study the in vitro simulation of digestion. The difference of the crude fat portion between dog food and pig feed means that previously reported protocols need to be modified if they are to be applied to studies with dogs [19]. There are only a few studies of static in vitro digestion models of crude fat, some of which suggest that pancreatic enzymes and bile salts are necessary to promote fat digestion [6]. The main role of pancreatin, which contains various digestive enzymes including lipase, is to digest fat, while bile salts emulsify fats that help lipase hydrolyze fats [16]. In the in vivo digestion process, bile salts were either re-absorbed, taken up by microbiota, or excreted [20]. On the other hand, those in vivo events did not happen during the in vitro digestion process. Therefore, it is important to obtain optimized concentrations of bile salts and pancreatin in in vitro digestion to establish an accurate model for the in vitro simulation of digestion. This study investigated the effects of bile salts and pancreatin on ether extract (EE), crude protein (CP), and dry matter (DM) in the in vitro digestion of dog food. The findings will contribute to the development of an optimized protocol for the in vitro digestion of dog food.

## 2. Materials and Methods

### 2.1. In Vivo Digestion

#### 2.1.1. Experimental Animals

This study was conducted on beagle dogs owned by the National Institute of Animal Science (NIAS). All animal experiments were performed in accordance with methods approved by the Animal Care and Use Committee, NIAS (NIAS-220-438). The experimental animal sample consisted of 24 neutered beagle dogs (12 females and 12 males). Dogs used in the present study had similar body weight (BW; 11.79 ± 0.49 kg, mean ± SD) and age (three-year-old) and were disease free, to avoid any untargeted effects on digestibility. The dogs were maintained in separate rooms (170 × 210 cm) at constant room temperature (22–23 °C) and lighting (16 h light and 8 h dark cycle). Food was provided twice daily (10:00 and 16:00), and an unlimited supply of drinking water was provided. The dogs were allowed approximately 6 h of outdoor activity per day. An extruded, pellet-type commercial feed (Iskhan All-life33, Wooriwa Ltd., Seoul, Korea) was provided at an amount that meets the maintenance energy requirements, which were estimated using the formula (ME, kcal/day; 132 × BW_0.75_ kg) presented by the Association of American Feed Control Officials (Table 1) [21].

#### 2.1.2. Apparent Total Tract Digestibility (ATTD)

The evaluation of ATTD was estimated using an indirect method using 0.5% Cr_2_O_3_. Feces, feed moisture, CP, and EE were analyzed according to the standard methods of the Association of Official Analytical Chemists [22]. Digestibility was calculated from the Cr_2_O_3_ concentration in the feces according to the following formula [23]:

(1) $$\mathrm{Digestibility}\;(\%)=100-(\frac{Cr\;input\left(food\right)\times Nutrient\;output\left(fecal\right)}{Cr\;output\left(fecal\right)\times Nutrient\;input\left(food\right)})\times 100$$

### 2.2. In Vitro Simulation of Digestion

#### 2.2.1. Static In Vitro Digestion Model

The in vitro digestion model consisted of two stages of digestion in the stomach and small intestine [6,16,24,25,26]. Digestion was carried out sequentially from the gastric phase to the small intestinal phase. This method can be briefly summarized as follows.

1. Sample preparation: We used an extruded commercial dog food that is identical to the one used for the in vivo digestion test. The sample was finely pulverized (<1 mm particle size) after being dried in an oven (65 °C) to a constant weight.
2. Gastric digestion phase: In Erlenmeyer glass flasks (500 mL), 5 g of dog food sample (<1 mm), 125 mL (25 mL/g feed) 0.1 M phosphate buffer (pH 6.0), and 50 mL (10 mL/g feed) 0.2 M HCl were added, and the pH was adjusted to 2.0 using 0.1 M HCl. We then added 1 mL pepsin–HCl (50 mg/mL 0.075 N HCl and pepsin from porcine gastric mucosa, ≥250 units/mg, P7000, Sigma Aldrich, St. Louis, MO, USA) and 1 mL chloramphenicol (2.5 mg/mL in ethanol, C-0378, Sigma Aldrich, St. Louis, MO, USA) solutions and incubated the mixture in a shaking water bath (39 °C, 130 rpm) for 6 h.
3. Small-intestinal phase: Once gastric digestion was completed, 50 mL (10 mL/g feed) 0.2 M phosphate buffer (pH 6.8) and 25 mL (10 mL/g feed) 0.6 M NaOH were added, and the pH was adjusted to 6.8 using 0.1 M NaOH. Thereafter, 6.250 g (100%, 25 g/L digestive solution), 3.125 g (50%, 12.5 g/L digestive solution), 1.563 g (25%, 6.25 g/L digestive solution), 0.625 g (10%, 2.5 g/L digestive solution), and 0 g (0%) of bile salts (13805, Sigma Aldrich, St. Louis, MO, USA) as well as 2.50 g (100%, 10 g/L digestive solution), 1.25 g (50%, 5 g/L digestive solution), 0.625 g (25%, 2.5 g/L digestive solution), 0.250 g (10%, 1 g/L digestive solution), and 0 g (0%) of pancreatin (P7545, 8 × USP specifications, Sigma Aldrich, St. Louis, MO, USA) were, respectively, added to each flask. This was incubated in a shaking water bath (39 °C, 150 rpm) for 18 h.
4. Collection of undigested fraction: The undigested fraction was collected using a bottle-top vacuum filter (pore size 0.22 µm, PES membranes, TPP), dried in a dry oven (65 °C), and then weighed.

#### 2.2.2. Calculation of In Vitro Digestibility 

The digestibility of DM in dog food was determined by weighing the undigested fraction after in vitro digestion obtained from each experimental group using the following equation [6]:

(2) $$\mathrm{DM}\;\mathrm{digestibility}\;(\%)=(\frac{food,\;g-undigested\;fraction,\;g}{food,\;g})\times 100$$

We followed the methods of the AOAC (2006) to measure the CP (AOAC method 984.13) and EE (AOAC method 920.39) contents in the undigested fraction, and their digestibility was calculated using the following equations [6]:

(3) $$\mathrm{CP}\;\mathrm{digestibility}\;(\%)=(\frac{food\;CP,\;g-undigested\;fraction\;CP,\;g}{food\;CP,\;g})\times 100$$

(4) $$\mathrm{EE}\;\mathrm{digestibility}\;(\%)=(\frac{food\;EE,\;g-undigested\;fraction\;EE,\;g}{food\;EE,\;g})\times 100$$

### 2.3. Statistical Analysis

The data were confirmed to not depart from normality significantly, using the Shapiro–Wilk method (*p* > 0.05). 

One-way analysis of variance was performed with the data obtained from each measurement using SPSS (version 17.0, SPSS Statistics, IL, USA, 2009), and significant difference between the means was analyzed at *p* < 0.05 with Tukey test. All data are expressed as mean ± standard error (SE). 

## 3. Results 

### 3.1. Apparent Total Tract Digestibility in Beagle Dogs

To determine the in vivo digestibility of a commercial dog food, we measured the ATTD of adult beagles (n = 24) using an indicator method with 0.5% chromium oxide. The ATTD of an adult beagle was 96.75% ± 0.21% for DM, 91.71% ± 0.62% for CP, 97.90% ± 0.23% for EE, 90.12% ± 0.91% for crude fiber, 74% ± 19% for crude ash, and 87.12% ± 0.82% for nitrogen-free extract. 

### 3.2. Static In Vitro Digestion for Dogs

#### 3.2.1. In Vitro EE Digestibility

**Figure 1:**
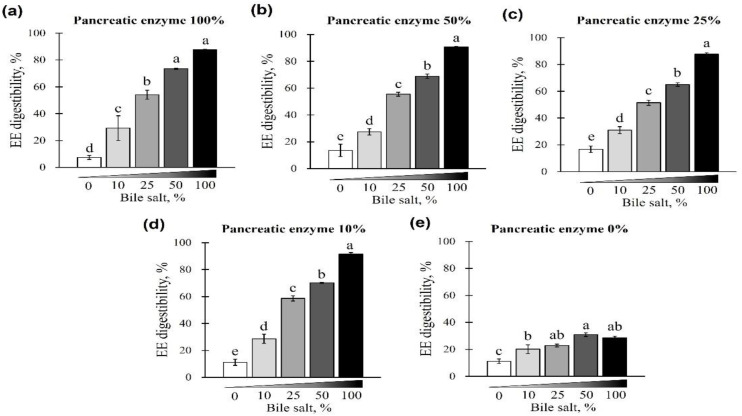
The effect of different concentrations of pancreatin and bile salts on the digestibility of ether extract (EE) in a commercial extruded dog food in in vitro digestion. Commercial extruded dog food digested with (**a**) 100% (10 g/L digestive solution) pancreatin and 0–100% (0–6.25 g/L digestive solution) bile salts; (**b**) 50% pancreatin (5 g/L digestive solution) and 0–100% bile salts; (**c**) 25% (2.5 g/L digestive solution) pancreatin and 0–100% bile salts; (**d**) 10% pancreatin (1 g/L digestive solution) and 0–100% bile salts; and (**e**) 0–100% bile salts only. The values are expressed as mean ± SEM from three independent experiments. ^a–e^ Different superscript letters denote significant differences among the experimental groups (*p* < 0.05).

**Figure 2:**
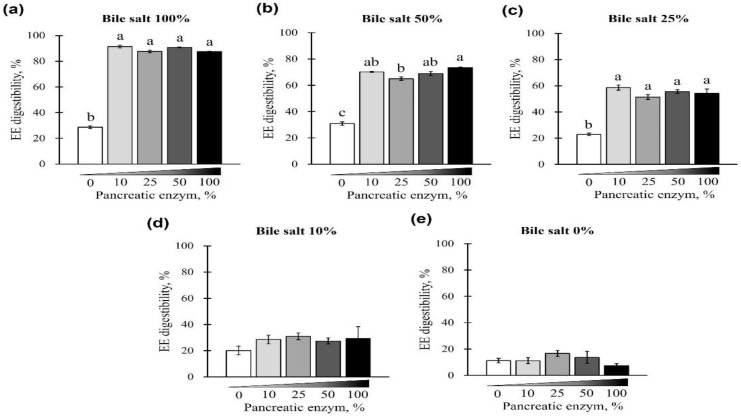
The effect of different concentrations of pancreatin and bile salts on the digestibility of EE in a commercial extruded dog food in in vitro digestion. Commercial extruded dog food digested with (**a**) 100% bile salts (25 g/L digestive solution) and 0–100% (0–10 g/L digestive solution) pancreatin; (**b**) 50% bile salts (12.5 g/L digestive solution) and 0–100% pancreatin; (**c**) 25% bile salts (6.25 g/L digestive solution) and 0–100% pancreatin; (**d**) 10% bile salts (2.5 g/L digestive solution) and 0–100% pancreatin; and (**e**) 0–100% pancreatin only. The values are expressed as mean ± SEM from three independent experiments. ^a–c^ Different superscript letters denote significant differences among the experimental groups (*p* < 0.05).

First, EE digestibility was evaluated based on different combinations of pancreatin and bile salt concentrations from 0 to 100%. A gradual increase in EE digestibility was observed at each pancreatin concentration (0, 10, 25, 50, and 100%) when bile salts were added from 0 to 100% (*p* < 0.05; Figure 1a–e). The digestibility of the EE was lowest when bile salts were not added to any of the pancreatin groups (*p* < 0.05; Figure 1a–e) and highest when 100% bile salts were added. This trend was observed regardless of the pancreatin concentrations, while no significant change in EE digestibility was observed with the addition of pancreatin (*p* < 0.05; Figure 2a). At 25, 50, and 100% bile salt concentration, EE digestibility was similar for any concentration of pancreatin added. However, when pancreatin was not added, EE digestibility significantly decreased (Figure 2a–c). At 0 and 10% bile salt concentrations, no significant difference in EE digestibility was observed in groups treated with different pancreatin concentrations (*p* > 0.05; Figure 2d,e). 

#### 3.2.2. In Vitro CP Digestibility

**Figure 3:**
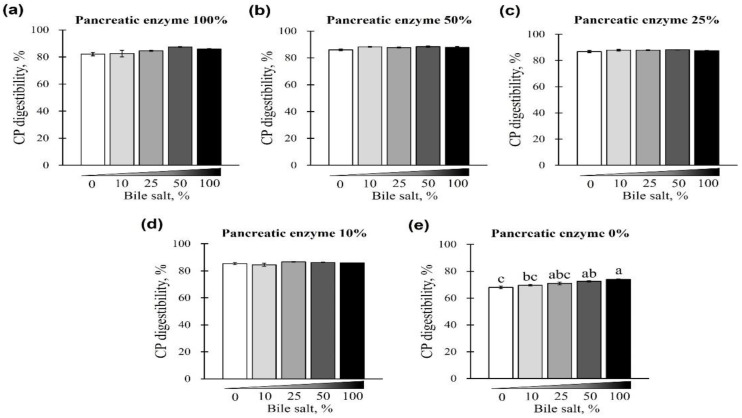
The effect of different concentrations of pancreatin and bile salts on the digestibility of crude protein (CP) in a commercial extruded dog food in in vitro digestion. Commercial extruded dog food digested with (**a**) 100% (10 g/L digestive solution) pancreatin and 0–100% bile salts (0–25 g/L digestive solution); (**b**) 50% pancreatin (5 g/L digestive solution) and 0–100% bile salts; (**c**) 25% pancreatin (2.5 g/L digestive solution) and 0–100% bile salts; (**d**) 10% pancreatin (1 g/L digestive solution) and 0–100% bile salts; and (**e**) 0–100% bile salts only. The values are expressed as mean ± SEM from three independent experiments. ^a–c^ Different superscript letters denote significant differences among the experimental groups (*p* < 0.05).

**Figure 4:**
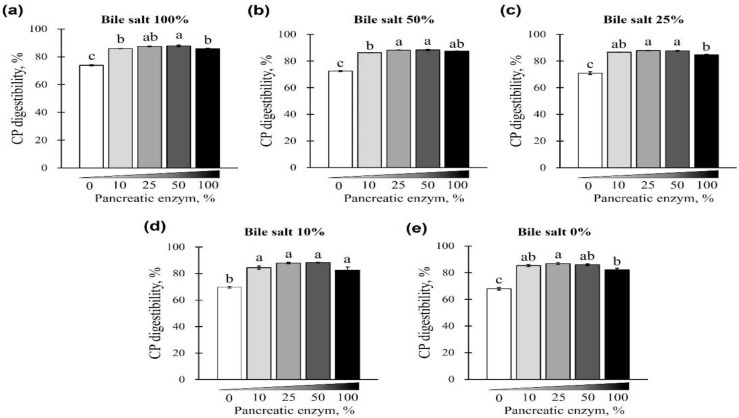
The effect of different concentrations of pancreatin and bile salts on the digestibility of CP in a commercial extruded dog food in in vitro digestion. Commercial extruded dog food digested with (**a**) 100% bile salts (25 g/L digestive solution) and 0–100% pancreatin (0–10 g/L digestive solution); (**b**) 50% bile salts (12.5 g/L digestive solution) and 0–100% pancreatin; (**c**) 25% bile salts (6.25 g/L digestive solution) and 0–100% pancreatin; (**d**) 10% bile salts (2.5 g/L digestive solution) and 0–100% pancreatin; and (**e**) 0–100% pancreatin only. The values are expressed as mean ± SEM from three independent experiments. ^a–c^ Different superscript letters denote significant differences among the experimental groups (*p* < 0.05).

The digestibility of CP using different concentrations of pancreatin and bile salts is shown in Figure 3 and Figure 4. The digestibility of CP was observed in all bile-salt-treated groups, with no differences based on the concentration of added bile salts (*p* > 0.05; Figure 3a–d). However, a CP digestibility of 73.94% ± 0.44% (maximum) and 67.97% ± 1.66% (minimum) was observed at 0% pancreatin when 0 or 100% bile salts were added, respectively. Furthermore, for all bile-salt-treated groups, the observed digestibility was 80% or less without the addition of pancreatin (*p* < 0.05, Figure 3e and Figure 4a–e). In the commercial dog food (CP, 37.5%) used in this study, CP digestibility was reduced in the absence of pancreatin, regardless of the amount of bile salts added (Figure 4a–e). 

#### 3.2.3. In Vitro DM Digestibility

**Figure 5:**
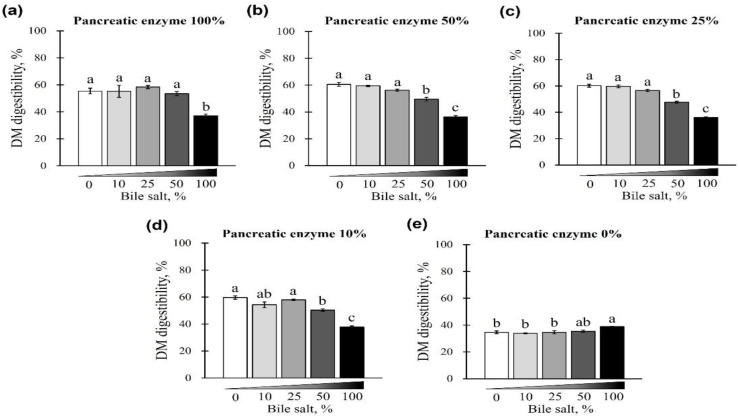
The effect of different concentrations of pancreatin and bile salts on the digestibility of dry matter (DM) in a commercial extruded dog food in in vitro digestion. Commercial extruded dog food digested with (**a**) 100% pancreatin (10 g/L digestive solution) and 0–100% bile salts (0–25 g/L digestive solution); (**b**) 50% pancreatin (5 g/L digestive solution) and 0–100% bile salts; (**c**) 25% pancreatin (2.5 g/L digestive solution) and 0–100% bile salts; (**d**) 10% pancreatin (1 g/L digestive solution) and 0–100% bile salts; and (**e**) 0–100% bile salts only. The values are expressed as mean ± SEM from three independent experiments. ^a–c^ Different superscript letters denote significant differences among the experimental groups (*p* < 0.05).

**Figure 6:**
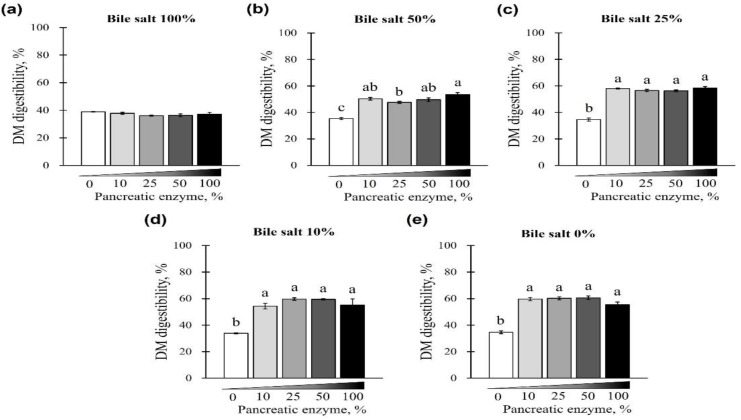
The effect of different concentrations of pancreatin and bile salts on the digestibility of DM in a commercial extruded dog food in in vitro digestion. Commercial extruded dog food digested with (**a**) 100% bile salts (25 g/L digestive solution) and 0–100% (0–10 g/L digestive solution) pancreatin; (**b**) 50% bile salts (12.5 g/L digestive solution) and 0–100% pancreatin; (**c**) 25% bile salts (6.25 g/L digestive solution) and 0–100% pancreatin; (**d**) 10% bile salts (2.5 g/L digestive solution) and 0–100% pancreatin; and (**e**) 0–100% pancreatin only. The values are expressed as mean ± SEM from three independent experiments. ^a–c^ Different superscript letters denoted significant differences among the experimental groups (*p* < 0.05).

The changes in DM digestibility with the addition of pancreatin and bile salts are shown in Figure 5 and Figure 6. The digestibility of DM was significantly reduced in groups treated with 50 and 100% concentrations of bile salts at 10–100% of pancreatin and was lowest at 100% bile salts (*p* < 0.05; Figure 5a–d). However, when bile salts were added at 0–100% without pancreatin, the digestibility of all groups reduced to <40% (*p* < 0.05; Figure 5e). Interestingly, when 0–100% pancreatin was used together with 100% bile salts, all experimental groups showed <40% DM digestibility with no observed changes owing to different concentrations of pancreatin (Figure 6a). In addition, when 10–100% pancreatin was used with 0–50% bile salts, the group without pancreatin showed the lowest digestibility, and no apparent change in digestibility was observed for groups with 10–100% pancreatin (Figure 6b–e).

## 4. Discussion

The in vitro simulation of digestion is an alternative and effective method of evaluating in vivo digestion indirectly. Although it has been used in pet food development, further methodological improvements are required. Therefore, in the present study, we investigated the digestive conditions of in vitro simulations of dog food digestion. Prior to the in vitro digestion experiment, ATTD was evaluated in beagle dogs. The level of digestibility was relatively higher than that reported earlier [27,28], probably owing to differences in types of ingredients (animal or plant-derived) [29,30], age of the dogs [31], and body type [32].

The results of static in vitro digestion study show that EE digestibility is influenced by the addition of pancreatin and bile salts. To date, static in vitro models simulating fat digestion are limited. In a particular study involving the static in vitro digestion model of dogs, similar to our results, high EE digestibility was observed upon the addition of bile salts at 25 g/L, regardless of the pancreatin concentrations (10 and 12.5 g/L) [6]. This is the only study that evaluated the effects of different concentrations of pancreatin and bile salts on EE digestibility. In addition, the observed fat digestion (>10% fat digestibility) despite the absence of pancreatin, which contains lipase, indicates the presence of an unexpected factor in the feed or buffer that influences EE digestion. Therefore, to infer in vitro digestibility accurately, analysis of the concentrations of pancreatin and bile salts and a consideration of the steps of a process (freezing, heating, or chemical treatment before in vitro digestion) that can eliminate unintended digestion reactions are both necessary.

Bile salts did not influence in vitro CP digestibility in the test under different concentrations of pancreatin and bile salts. However, ≤10% pancreatin can affect the digestibility of protein. Pancreatin contains various digestive enzymes, including proteases and proteolytic enzymes, which may explain its digestive effects [33]. To control CP digestibility, the concentration of added pancreatic enzymes in the small-intestinal phase should be considered along with the amount of pepsin added during the gastric digestion phase.

Next, DM digestibility was affected by bile salts. Pancreatin did not affect the DM digestibility when bile salts were applied at concentrations of 0–25%. The digestibility of DM was significantly reduced when >50% concentrations of bile salts were added with 10%–100% pancreatin. In addition, when bile salts were not added, a DM digestibility of approximately 60% was maintained, although EE digestibility decreased to less than 20%, which might increase DM digestibility. Bile salts are essential for fat emulsification and the formation of mixed micelles that solubilize and transport lipophilic products to the gut wall for absorption [16]. Furthermore, bile salts are reabsorbed into the body during digestion and can be lost owing to absorption by microorganisms or excretion [20]. Additionally, bile salts may be excreted in combination with dietary fibers [34]. During the digestive process in the body, the substrate-to-enzyme ratio changes owing to the digestion and absorption of substrates, whereas static in vitro digestion models do not include the process of removal (absorption) of enzymes or bile salts [16]. Such differences in digestive environments should be considered in the in vitro simulation of digestion process. Therefore, the appropriate concentrations of bile salts should be investigated further to facilitate the accurate estimation of digestibility.

Overall, the digestibility of a commercially extruded dog food analyzed by the static in vitro digestion model of our study (EE digestibility, 91.50% ± 1.11%; CP digestibility, 88.34% ± 0.58%; and DM digestibility, 60.61% ± 1.40%) was relatively lower than that of the ATTD in beagles (EE digestibility, 97.90% ± 0.23%; CP digestibility, 91.71% ± 0.62%; and DM digestibility, 96.75% ± 0.21%). Such differences may be due to the absorption of nutrients or fermentation of DM by intestinal microorganisms during the digestion process in the ileum and the residual bile salts in the undigested contents of a static in vitro digestion model. In addition, the results showed that pancreatin and bile salts affect the digestibility of DM, CP, and EE. Bile salt, particularly, affected the digestibility of EE in a concentration-dependent manner. The lowest digestibility of EE observed was 10%, even when no pancreatin and bile salts were added. Therefore, it can be implied that there could be a non-specific effect, such as loss of the digested phase during filtration. For more accurate in vitro digestibility, any unwanted effect on digestibility during in vitro processes should be minimized; for instance, by removing non-specific digestive reactions (freezing, heating, chemical treatment, etc.) or improving the harvesting technique of the digested fraction. Thus far, various in vitro digestion models for mimicking in vivo models have been suggested that are not restricted to nutrient digestibility research [12,13,35,36], medicine [37], toxicity [38], or plant-derived compounds [14,39] for application in biological evaluations. However, in vitro simulation models for dogs are few. The findings of the present study could facilitate the development of accurate, static in vitro models, especially for the digestion of EE in dog food. 

## 5. Conclusions

The results of the present study highlight the importance of digestive enzymes during static in vitro simulation to mimic in vivo digestion and identify the potential optimal concentrations of pancreatin and bile salts required to digest crude proteins, fats, and dry matter. CP and DM digestibility was reduced significantly without pancreatin and EE digestibility was reduced significantly when there was no bile salts. Pancreatin and bile salts are crucial factors influencing the in vitro simulation of digestion in dog food, which contains higher proportions of crude proteins and crude fats. Although improvements are still required, the suggested conditions of the static in vitro digestion model could facilitate the development of a standard protocols for the in vitro digestion of dog food.

## Figures and Tables

**Table 1:** Analyzed chemical composition of the experimental diet.

Items	Experimental Diet
Moisture, %	12
Crude Protein, %	37.5
Ether Extract, %	22.7
Crude Fiber, %	3.41
Crude Ash, %	14.8
Ca, %	1
P, %	0.8
Metabolic Energy, kcal/kg	4100

Values are calculated on dry matter basis. Ca, calcium; P, phosphorus.

## Data Availability

Not applicable.

## References

[1] Corsato Alvarenga I.C., Aldrich C.G. (2019). The Effect of Increasing Levels of Dehulled Faba Beans (*Vicia faba* L.) on Extrusion and Product Parameters for Dry Expanded Dog Food. Foods.

[2] McCusker S., Buff P.R., Yu Z., Fascetti A.J. (2014). Amino Acid Content of Selected Plant, Algae and Insect Species: A Search for Alternative Protein Sources for Use in Pet Foods. J. Nutr. Sci..

[3] Franco N.H. (2013). Animal Experiments in Biomedical Research: A Historical Perspective. Animals.

[4] Hampshire V.A., Gilbert S.H. (2019). Refinement, Reduction, and Replacement (3R) Strategies in Preclinical Testing of Medical Devices. Toxicol. Pathol..

[5] Kerecman Myers D.K., Goldberg A.M., Poth A., Wolf M.F., Carraway J., McKim J., Coleman K.P., Hutchinson R., Brown R., Krug H.F. (2017). From In Vivo to In Vitro: The Medical Device Testing Paradigm Shift. ALTEX.

[6] Biagi G., Cipollini I., Grandi M., Pinna C., Vecchiato C.G., Zaghini G. (2016). A New In Vitro Method to Evaluate Digestibility of Commercial Diets for Dogs. Ital. J. Anim. Sci..

[7] Vors C., Capolino P., Guérin C., Meugnier E., Pesenti S., Chauvin M.A., Monteil J., Peretti N., Cansell M., Carrière F. (2012). Coupling In Vitro Gastrointestinal Lipolysis and Caco-2 Cell Cultures for Testing the Absorption of Different Food Emulsions. Food Funct..

[8] Theysgeur S., Cudennec B., Deracinois B., Perrin C., Guiller I., Lepoudère A., Flahaut C., Ravallec R. (2020). New Bioactive Peptides Identified from a Tilapia Byproduct Hydrolysate Exerting Effects on DPP-IV Activity and Intestinal Hormones Regulation after Canine Gastrointestinal Simulated Digestion. Molecules.

[9] Wickham M., Faulks R., Mills C. (2009). In Vitro Digestion Methods for Assessing the Effect of Food Structure on Allergen Breakdown. Mol. Nutr. Food Res..

[10] Ménard O., Cattenoz T., Guillemin H., Souchon I., Deglaire A., Dupont D., Picque D. (2014). Validation of a New In Vitro Dynamic System to Simulate Infant Digestion. Food Chem..

[11] Maldonado-Valderrama J., Gunning A.P., Wilde P.J., Morris V.J. (2010). In Vitro Gastric Digestion of Interfacial Protein Structures: Visualisation by AFM. Soft Matter.

[12] Hasjim J., Lavau G.C., Gidley M.J., Gilbert R.G. (2010). In Vivo and In Vitro Starch Digestion: Are Current In Vitro Techniques Adequate?. Biomacromolecules.

[13] Larsson K., Cavonius L., Alminger M., Undeland I. (2012). Oxidation of Cod Liver Oil During Gastrointestinal In Vitro Digestion. J. Agric. Food Chem..

[14] Biehler E., Bohn T. (2010). Methods for Assessing Aspects of Carotenoid Bioavailability. Curr. Nutr. Food Sci..

[15] Lorrain B., Dangles O., Loonis M., Armand M., Dufour C. (2012). Dietary Iron-Initiated Lipid Oxidation and Its Inhibition by Polyphenols in Gastric Conditions. J. Agric. Food Chem..

[16] Minekus M., Alminger M., Alvito P., Ballance S., Bohn T., Bourlieu C., Carrière F., Boutrou R., Corredig M., Dupont D. (2014). A Standardised Static In Vitro Digestion Method Suitable for Food—An International Consensus. Food Funct..

[17] Kazimierska K., Biel W., Witkowicz R., Karakulska J., Stachurska X. (2021). Evaluation of Nutritional Value and Microbiological Safety in Commercial Dog Food. Vet. Res. Commun..

[18] Liu Y., Kil D.Y., Perez-Mendoza V.G., Song M., Pettigrew J.E. (2018). Supplementation of Different Fat Sources Affects Growth Performance and Carcass Composition of Finishing Pigs. J. Anim. Sci. Biotechnol..

[19] Piccione G., Fazio F., Giudice E., Grasso F., Caola G. (2004). Blood Lipids, Fecal Fat and Chymotrypsin Excretion in the Dog: Influence of Age, Body Weight and Sex. J. Vet. Med. Sci..

[20] Naumann S., Haller D., Eisner P., Schweiggert-Weisz U. (2020). Mechanisms of Interactions Between Bile Acids and Plant Compounds-A Review. Int. J. Mol. Sci..

[21] Association of American Feed Control Officials (AAFCO) (2016). Model Bill and Regulations.

[22] Association of Official Analytical Chemists (AOAC) (2006). Official Methods of Analysis of AOAC International.

[23] Kim K.H., Seo K., Cho H.W., Jeon J.H., Kim C.H., Jung J., Chun J.L. (2021). Age-Related Digestibility of Nutrients Depending on the Moisture Content in Aged Dogs. J. Anim. Sci. Technol..

[24] Boisen S., Ferna’ndez J.A. (1995). Prediction of the Apparent Ileal Digestibility of Protein and Amino Acids in Feedstuffs and Feed Mixtures for Pigs by In Vitro Analyses. Anim. Feed Sci. Technol..

[25] Cianciosi D., Forbes-Hernández T.Y., Afrin S., Gasparrini M., Quiles J.L., Gil E., Bompadre S., Simal-Gandara J., Battino M., Giampieri F. (2020). The Influence of In Vitro Gastrointestinal Digestion on the Anticancer Activity of Manuka Honey. Antioxidants.

[26] Doumani N., Severin I., Dahbi L., Bou-Maroun E., Tueni M., Sok N., Chagnon M.C., Maalouly J., Cayot P. (2020). Lemon Juice, Sesame Paste, and Autoclaving Influence Iron Bioavailability of Hummus: Assessment by an In Vitro Digestion/Caco-2 Cell Model. Foods.

[27] Ahlstrom O., Skrede A. (1998). Comparative Nutrient Digestibility in Dogs, Blue Foxes, Mink and Rats. J. Nutr..

[28] Daumas C., Paragon B.M., Thorin C., Martin L., Dumon H., Ninet S., Nguyen P. (2014). Evaluation of Eight Commercial Dog Diets. J. Nutr. Sci..

[29] Cargo-Froom C.L., Fan M.Z., Pfeuti G., Pendlebury C., Shoveller A.K. (2019). Apparent and True Digestibility of Macro and Micro Nutrients in Adult Maintenance Dog Foods Containing Either a Majority of Animal or Vegetable Proteins1. J. Anim. Sci..

[30] Kahraman O., İnal F. (2021). Comparison of Digestibility Parameters of Commercial Dry Dog Foods with Different Contents. Arq. Bras. Med. Vet. Zootec..

[31] Schauf S., Stockman J., Haydock R., Eyre R., Fortener L., Park J.S., Bakke A.M., Watson P. (2021). Healthy Ageing Is Associated with Preserved or Enhanced Nutrient and Mineral Apparent Digestibility in Dogs and Cats Fed Commercially Relevant Extruded Diets. Animals.

[32] Weber M.P., Biourge V.C., Nguyen P.G. (2017). Digestive Sensitivity Varies According to Size of Dogs: A Review. J. Anim. Physiol. Anim. Nutr..

[33] Wu Y.Y., Ding L., Xia H.L., Tu Y.Y. (2010). Analysis of the Major Chemical Compositions in Fuzhuan Brick-Tea and Its Effect on Activities of Pancreatic Enzymes In Vitro. Afr. J. Biotechnol..

[34] Singh J., Metrani R., Shivanagoudra S.R., Jayaprakasha G.K., Patil B.S. (2019). Review on Bile Acids: Effects of the Gut Microbiome, Interactions with Dietary Fiber, and Alterations in the Bioaccessibility of Bioactive Compounds. J. Agric. Food Chem..

[35] Kopf-Bolanz K.A., Schwander F., Gijs M., Vergères G., Portmann R., Egger L. (2012). Validation of an In Vitro Digestive System for Studying Macronutrient Decomposition in Humans. J. Nutr..

[36] Miller D.D., Schricker B.R., Rasmussen R.R., Van Campen D. (1981). An In Vitro Method for Estimation of Iron Availability from Meals. Am. J. Clin. Nutr..

[37] Porter C.J., Kaukonen A.M., Boyd B.J., Edwards G.A., Charman W.N. (2004). Susceptibility to Lipase-Mediated Digestion Reduces the Oral Bioavailability of Danazol after Administration as a Medium-Chain Lipid-Based Microemulsion Formulation. Pharm. Res..

[38] Versantvoort C.H., Oomen A.G., Van de Kamp E., Rompelberg C.J., Sips A.J. (2005). Applicability of an In Vitro Digestion Model in Assessing the Bioaccessibility of Mycotoxins from Food. Food Chem. Toxicol..

[39] Bouayed J., Hoffmann L., Bohn T. (2011). Total Phenolics, Flavonoids, Anthocyanins and Antioxidant Activity Following Simulated Gastro-intestinal Digestion and Dialysis of Apple Varieties: Bioaccessibility and Potential Uptake. Food Chem..

